# Effects of stimulus amplitude-scaling approach on emotional responses to non-speech sounds

**DOI:** 10.1371/journal.pone.0328659

**Published:** 2025-07-31

**Authors:** Erin M. Picou, Shae D. Morgan, Elizabeth D. Young, Samantha J. Gustafson

**Affiliations:** 1 Department of Hearing and Speech, Vanderbilt University Medical Center, Nashville, Tennessee, United States of America; 2 Program in Audiology, University of Louisville, Louisville, Kentucky, United States of America; 3 Department of Communication Sciences and Disorders, University of Utah, Salt Lake City, Utah, United States of America; Max Planck Institute for Empirical Aesthetics: Max Planck Institut fur empirische Asthetik, GERMANY

## Abstract

In the study of auditory emotion perception, it is important to calibrate test sounds so their presentation level during testing is known. It is also often desirable to standardize the amplitude of the sounds so that each sound used in testing is approximately the same level. However, existing literature in the study of auditory emotion perception includes a mixture of techniques for standardizing amplitude across sounds. The purpose of this study was to compare the effects of two amplitude-scaling approaches on emotional responses to non-speech sounds, specifically standardization based on peak level or root-mean-square (rms) level. Nineteen young adults provided ratings of valence and arousal via an online testing program. Stimuli were non-speech sounds scaled in two ways, based on the stimulus’ peak level or rms level. Ratings were analyzed using linear-mixed effects modeling to compare scaling methods; correlations between ratings and level within each scaling method were explored. Analysis revealed that the ratings of peak-scaled sounds were less pleasant and more exciting than were the ratings of rms-scaled sounds, although the effects were small in magnitude (~0.2 points on a 1–9 scale). Within rms-scaled sounds, peak level was not related to ratings of valence or arousal. However, within peak-scaled sounds, rms level was related to ratings of valence and arousal. Combined, these data suggest that amplitude standardization has a small effect on ratings overall, but investigators might be motivated to choose one approach over the other, depending on the research question. Rms-scaling reduces overall level as a cue for emotional responses, while peak-scaling maintains some natural variability in responses related to level. Finally, results are specific to this stimulus set. The effects of amplitude-scaling would be expected to be negligible for a stimulus set where the sounds have homogenous temporal dynamics.

## Introduction

Emotions play an important part of daily life experiences, facilitating cognition, attention, stress recovery, and well-being [1–6]. Investigations using a variety of paradigms have shown that perception of emotion is atypical in several populations, including those with brain damage [7,8], neurodegenerative diseases [e.g., 9–11], pragmatic language deficits [e.g., 12–14], and hearing loss [4,15,16]. Therefore, it is important to understand the conceptual and methodological factors that affect measurement of emotion perception.

The dimensional view of emotion provides a framework for studying emotion. Under the dimensional view, emotions are conceptualized as a combination of two or more continua, most often hedonistic valence (pleasant, unpleasant) and arousal (exciting, calming); valence represents the direction of the emotion and arousal represents the degree of activation of the emotion [17–19]. One example of how this dimensional view can inform our understanding of emotion’s role in communication disorders is evident in listeners with hearing loss. Using this framework, there have been several investigations into emotional responses to sounds for adults with and without hearing loss using ratings of valence and arousal. The results consistently indicate that people with hearing loss demonstrate a reduced range of valence, with valence ratings less extreme (less pleasant and less unpleasant) than their peers’ with normal hearing; similar effects are not often found for arousal [20–23].

In addition to their usefulness in studying experiences of individuals with hearing loss, emotionally evocative stimuli also have the potential to interfere with communication by distracting listeners. Novel sounds, even irrelevant ones, are processed automatically and rapidly [24]. These irrelevant sounds can capture attention, distracting listeners from their intended auditory target and impairing target recognition [25–29]. For example, a barking dog can distract from a conversation, affecting communication. Importantly, the extent to which irrelevant emotional stimuli distract from target speech depends upon the characteristics of that stimulus, including their perceived valence or arousal. For example, pleasant and unpleasant sounds have been shown to be more distracting than neutral sounds [30].

While this dimensional framework is being used to evaluate groups of individuals and to target specific processing mechanisms, surprisingly little is known about how stimulus calibration choices influence the subjective ratings of these emotional dimensions. To better answer questions surrounding emotional auditory distraction, or even to continue investigating emotion perception for people with hearing loss or other communication disorders, it is important to evaluate the degree to which methodological choices result in generalizable results. The focus of this project is the comparison of two amplitude-standardization approaches on ratings of valence and arousal for non-speech sounds.

### Amplitude scaling

The choice of sound standardization, or amplitude scaling, could be critically important in the study of auditory emotion perception. Recent literature suggests that differences in level can influence environmental sound recognition [31] and emotional responses to non-speech sounds [20,21]. For example, Picou et al. [21] found that a 20 dB increase in overall level reduced ratings of valence in adults with and without hearing loss. However, the corpus of sounds often used for evaluation of emotional responses to non-speech sounds, the International Affective Digitized Sounds (IADS-2) corpus [32], consists of sounds that vary in peak and mean amplitudes. Furthermore, some studies of auditory distraction lack methodological descriptions surrounding stimulus amplitude, with no mention of amplitude scaling [33–35], as do many published studies on the emotional responses to non-speech sounds for people with hearing loss or tinnitus [36–39].

While some investigators chose not to control stimulus amplitude, others manipulate the sounds by scaling them based on their root-mean-square (rms) level [24,40–46]. This rms amplitude-scaling approach has a longstanding tradition in hearing science with speech sounds as experimental stimuli, where investigators match the rms level of all speech sounds (or segments or sentences) in a given condition [47–52]. This approach is well-suited for speech sounds, where the speech segments have predictable and relatively stable temporal dynamics, owing to the shared sound source (vocal tract) [53]. However, non-speech sounds are heterogeneous in terms of temporal dynamics [54], with no shared sound source and reflecting a wide range of types of sounds (e.g., music, animal sounds, body noises) [53].

**Fig 1:**
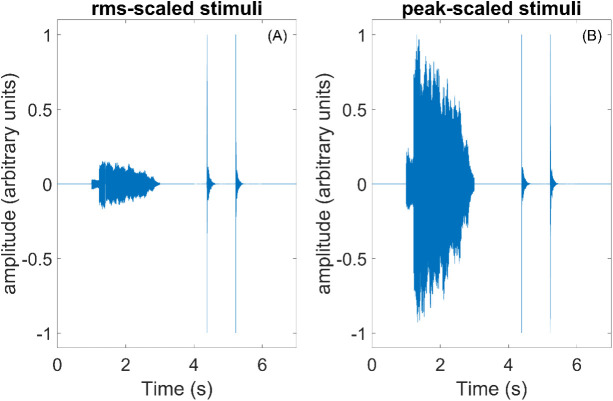
Examples of (A) rms- and (B) peak-scaled sound samples. The figure visually highlights differences between methodologies. Within each plot, constant (“ah” vocalization; left) and intermittent (two hand claps; right) time waveforms are displayed.

Despite the increased variability in temporal dynamics of non-speech sounds, some investigators have reported using the rms amplitude-scaling approach for studies with non-speech stimuli [e.g., 55–57]. The potential disadvantage of rms-scaling across sounds is a homogenization of temporal dynamics across stimuli. For example, if a non-speech sound has several long, low-intensity portions interspersed between periods of higher intensity (e.g., clapping), then the low-amplitude segments will strongly influence the rms amplitude, yielding a sound that may be perceptually louder than reference sounds with the same rms amplitude, but a more constant amplitude (e.g., steady vocalization; see left panel Fig 1).

**Fig 2:**
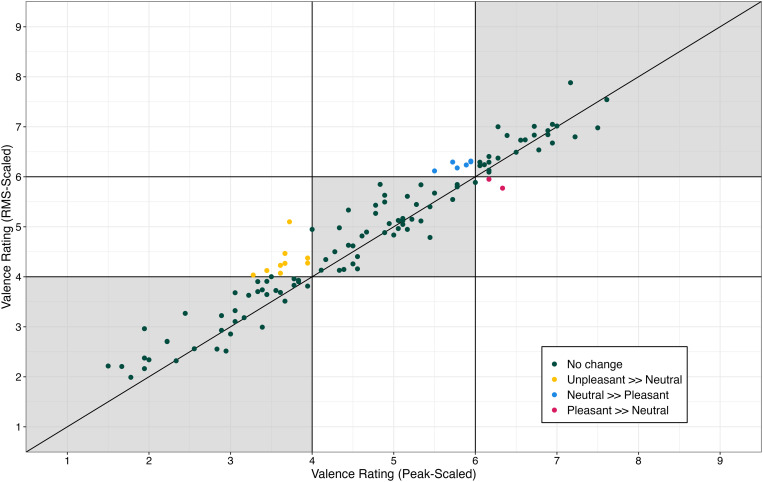
Mean ratings of valence of sounds when they were rms-scaled as a function of ratings of valence when tokens were peak-scaled. Each dot represents an individual sound. Shaded squares indicate ratings that did not change nominal category between scaling approaches. Yellow dots indicate sounds that were nominally ‘unpleasant’ with the peak-scaling approach but were nominally ‘neutral’ with the rms-scaling approach. Blues dots indicate sounds that were nominally ‘neutral’ with the peak-scaling approach but were ‘pleasant’ with the rms-scaling approach. Red dots indicate tokens that were nominally ‘pleasant’ with the peak-scaling approach but were nominally ‘neutral’ with the rms-scaling approach.

Another popular approach to controlling stimulus amplitude is peak scaling, where the peak outputs are matched across sounds (see right panel Fig 2). Maintaining variability in level across sounds might be useful for non-speech sounds, where real-world variability is expected. For example, the IADS corpus includes nature sounds, social sounds, and machine noises, which would naturally be expected to vary in rms amplitude in the real world. In addition, maintaining some variability in rms level might be useful because temporal dynamics are useful for categorizing non-speech sounds [54,58]. In addition, overall level has been shown to be related to emotional responses, specifically ratings of valence [20,21] and arousal [59–63]. Therefore, it is possible that an rms-scaling approach would reduce the usability of one of the cues (level) useful for emotion perception.

The use of peak scaling has been reported in several investigations of vocal emotion recognition [e.g., 64, 65], as well as studies using non-speech sounds for auditory distraction [25,66] and for the study of emotional responses [20–23,67]. It is not clear what effect, if any, the difference in amplitude-scaling strategies across studies might have on emotional responses to non-speech sounds or whether the amplitude-scaling approaches reduce level cues used for emotion perception. Among the studies where amplitude-scaling approach is described, none of the authors offer justification for their scaling approach [e.g., 21, 55–57], except to say their approach is consistent with previous work [20,22,67]. Although there exist accepted scaling approaches in other domains, such as the loudness unit referenced to full scale (LUFS) used in broadcasting [68], there are not currently recommendations for standardizing non-speech sounds for the purpose of evaluating emotion perception.

### Purpose

The purpose of this study was to evaluate the effects of amplitude-scaling approach (rms, peak) on emotional responses, specifically ratings of valence and arousal. The results of this study will inform future work with regards to the amplitude-scaling approach that could affect the study of emotional responses to sounds, as well as provide additional context for the interpretation of previous work using both approaches. Should one of the amplitude-scaling approaches result in significantly different ratings, it would provide evidence that the choice of scaling approaches might affect the interpretation of results and therefore confound the synthesis of findings across studies with differing amplitude-scaling approaches. In addition, the relationship between ratings of valence (or arousal) and level within an amplitude-scaling approach was explored to determine if one of the scaling approaches allowed for level to serve as a cue for emotion perception. The finding that one scaling approach reduced or preserved the relationship between ratings and level would also be informative for researchers when considering study designs. Finally, relationships between ratings of valence and level are generally informative in that the results could potentially provide insight into an acoustic cue for ratings of valence and/or arousal.

## Materials and methods

### Participants

Twenty-two adults (3 male and 19 female) participated. An additional 2 potential participants started but did not finish the study because they failed the headphone or browser requirements (detailed below). Participants reported to be white (**n* *= 16), Asian (*n* = 2), Hispanic (*n* = 1), or more than one race (**n* *= 3). They were recruited through word of mouth within undergraduate and graduate school communities at three universities. Participants were included if they self-reported to be native English speakers, to not have diagnosed psychological disorders (e.g., clinical depression), or be taking psychotropic medications (e.g., antidepressants). They reported normal hearing sensitivity, and their median self-reported hearing ability was 9, in response to the question, “On a scale of 1 to 10, how would you rate your hearing? 10 indicates excellent hearing.” All responses to this question were 8 or higher. They participated anonymously via an on-line experiment building and hosting software [Gorilla; 69] and were paid via e-mailed gift certificate. The study was conducted with the approval of the Vanderbilt University Medical Center Institutional Review Board (#211513).

### Stimuli

The valence and arousal dimensions of the self-assessment manikin [17] were used to facilitate ratings of emotional response to sounds. The dimensions each include five figures displaying a range of emotion along the dimension, specifically a smiling face to a frowning face for valence and an excited person to a sleepy person for arousal. Under each set of pictures was a slider, where whole numbers appeared when the participant moved the slider (with 1 and 9 at the extremes indicating low or high rating on either dimension). The numerical rating options (numbers 1–9) were equally spaced under the 5 manikin images. The sliders always started in the middle (rating of 5) and both had to be moved (or at least clicked) before advancing to the next trial.

Two sets of non-speech sounds were included: 1) a set of sounds that were the primary focus of this study (hereafter referred to as ‘study sounds’), which were previously used to evaluate auditory distraction [30] and 2) a previously validated set that has published normative data available (hereafter referred to as ‘validation sounds’). Stimuli that were previously validated served as a control for our listeners. This allowed us to ensure that their performance was consistent with established data using previously validated stimuli and, if so, would lend support that their ratings of study sounds reflect a typical population of young adults with normal hearing. The validation sounds consisted of sounds from the International Affective Digitized Sounds corpus [IADS2; 32]. The validation sounds in the current study were a subset of nine sounds from those used previously [23]. The validation sounds had normative ratings of valence ranging from 2.06 to 7.78, reflecting sounds that elicit a wide range of ratings of valence. The IADS tokens used were: 226 (laughing), 351 (applause), 817 (bongos), 120 (rooster), 410 (helicopter), 425 (train 255 (vomit), 295 (sobbing), and 296 (crying). The IADS were 6.0 seconds in duration.

The study sounds included 120 environmental sounds [33], that included sounds like throat clearing, aluminum can opening. The sounds were previously trimmed to be 0.5 s in duration by Marcell and colleagues [24]. The study sounds were chosen because they were used previously in a study of auditory distraction [29,30] and because they were expected to elicit a range of valence and arousal responses.

For both sets of sounds (study sounds and validation sounds), two sets of stimuli were created, one set where the sounds were all matched to have the same peak level (−3.01 dB relative to a maximum value of 1) or the same rms level (−26.9 dB relative to a maximum value of 1). Consistent with work in the area [e.g., 21, 23, 67], the peak level of –3.01 dB was chosen to preserve sufficient headroom to provide a clear signal without risk of peak-clipping and its associated distortion. All amplitude scaling was accomplished via custom MATLAB script (version 2021a). Relative to the maximum of 1, mean rms level for the peak-scaled sounds was –14.7 dB (standard deviation [*SD*] rms dB = 2.29; mean rms voltage = .19, *SD* rms voltage = .06). Relative to a maximum of 1, the mean peak level for the rms-scaled sounds was –15.2 dB (*SD* peak dB = 2.29; mean peak voltage = .18, *SD* peak voltage = .04). The actual stimulus presentation level and relative level for the two types of amplitude scaling approaches depended on the volume adjustments by the individual participants during testing (see below for details).

### Procedures

Due to the ongoing COVID-19 pandemic and because the tests of emotion perception can be reasonably accomplished using remote procedures [23,70,71], data collection in this study occurred remotely. Participants who indicated interest were sent a link to an on-line survey hosted in REDCap [72], which include brief demographic questions, informed consent document, and contact information for research personnel. Once participants provided electronic, written consent, they were directed to a new web address for the study procedures (hosted by Gorilla). Participants were required to use personal headphones or earphones during the experiment and to use a laptop or desktop computer using an HTML-5 compatible browser to complete the experiment. HTML-5 compatibility was required to ensure the browser would be able to play sound files in an uncompressed (lossless) format (i.e., *.wav). These three requirements were verified by the Gorilla software. Participants were also asked to perform the experiment in a quiet place, free from distractions, and to complete the experiment within one sitting. These requirements were not verified by the software; participants were held in good faith to complete them.

The study procedures began with a brief overview and introduction to the experiment.

Participants then performed a headphone test to ensure the participant was using one headphone in each ear. The headphone test was based on the work of Woods and colleagues [73], where participants are asked to discriminate the intensity of several tones. The tones were presented with phase differences of 180 degrees between the two channels. The task is designed to be easily achieved with headphones, but difficult through loudspeakers due to imperfect phase-cancellations of loudspeakers. The headphone check included six trials and participants were required to pass five of six trials for participation to continue.

The participant was then given instructions for the task (“For this experiment you will hear a number of sounds. We would like you to rate how excited/calm and how pleasant/unpleasant each sound makes you feel”) and a single practice trial consisting of a pleasant IADS-2 sound that was not later used for testing (a sample of music, IADS sound number 810). Prior to each testing block (described below), participants were asked to play the same IADS sound used in the familiarization block and to adjust their computer volume to a comfortable listening level (“You should be able to hear the sound, but it should not be too loud. Choose a level that is ‘just right’”). The sound used for volume testing was scaled to match the test sounds in the upcoming block (peak- or rms-scaled). The sound used for calibration had the approximately the same peak- and rms-level as the average of all the tokens in the peak- and rms-scaled conditions (rms level = −26.9 dB and peak = −16.1 dB in the rms-scaling approach and rms level = −13.9 dB and peak level = −3.01 dB in the peak-scaling approach, all relative to a maximum of 1).

After calibration, participants were asked not to change the volume for the remainder of the testing block. Participants then rated 129 non-speech sounds that were all rms- or peak-scaled (120 study sounds, 9 validation sounds). The sounds were presented in a random order, which effectively mixed stimuli of various durations (0.5 sec for study sounds and 6 sec for validation sounds) and expected emotional valence and arousal for a given amplitude-scaling approach inside of each block. At the halfway point of each testing block, participants were given a brief “attention check” where they were asked to adjust the sliders to a specific position (e.g., “Please move the Excitement slider all the way to the left and the Pleasantness slider all the way to the right”). Note that participants rated an additional 9 validation sounds in each amplitude-scaling condition to explore a different methodological question outside the scope of this project. Those data are not reported here.

Sounds were blocked by amplitude-scaling approach (i.e., peak-scaling vs rms-scaling). Blocks were counterbalanced between participants to account for block order effects that may exist. Between blocks, participants were instructed to take a break. Breaks of at least 1 minute were enforced by locking the experimental program and not allowing participants to proceed until the break time had passed. Before starting the next block, participants re-adjusted their volume to find a comfortable level using sound that had the same amplitude-scaling approach as the test sounds (e.g., rms-scaling). Following the rating of all sounds in both blocks, participants were then asked to disclose whether they had adjusted their volume after the calibration for each block. It was stressed that their honest answer would not affect compensation and would only be used to better understand the data. Finally, participants were directed back to REDCap and were invited to provide their e-mail address for receiving study compensation. Demographic information and contact information were not linked. Total test time was less than 60 minutes.

### Data analysis

All analyses were conducted in R [v. 4.3.0; [74]]. Three participants failed attention checks put in place during the remote testing; their data were excluded from analyses. All remaining participants (**n* *= 19) affirmed that they had not adjusted their volume after calibration in each testing block. Data analysis included multiple steps. First, a normative data check was accomplished to verify that participants’ responses were consistent with normative data of the validation sounds, thereby validating their understanding of the task and the reliability of their responses to the other study sounds (which lack normative validation). To perform this check, participants’ mean ratings of valence and arousal in response to the 9 validation sounds were compared to the mean normative ratings of valence and arousal available for the same sounds. One participant’s ratings of valence fell more than 1 standard deviation away from the normative values. Their mean valence rating was 3.17, whereas the mean valence rating in the normative data was 5.04 (*SD* = 1.65). Because their data were different than expected based normative data, the participant’s data were removed for the remaining analyses.

The effects of amplitude-scaling approach on ratings of valence and arousal were examined for the remaining 18 participants. To do this, ratings of valence and arousal were analyzed using linear-mixed effects modelling. Specifically, for each type of rating (valence, arousal), a linear mixed-effects model was constructed using the *lmer* function of the **lme4** package [75]. Each model included a single fixed factor, amplitude-scaling approach (rms, peak), in addition to random intercepts of participant and sound token. Models were analyzed and partial eta squared values were extracted using the *anova_stats* function of the **sjstats** package [76]. Significant main effects and interactions were explored using the *emmeans* function of the **emmeans** package [77] and controlling for false discovery rates [78].

To evaluate the effect of amplitude-scaling approach on ratings of valence or arousal for individual sounds, the number of sounds whose ratings of valence (or arousal) changed nominal category between the two scaling approaches was calculated. The purpose of this analysis was to evaluate if any changes in ratings would affect the interpretation of the rating; does a sound that elicits a ‘pleasant’ rating with an rms-scaling approach now elicit an ‘unpleasant’ rating with a peak-scaling approach? To evaluate nominal changes in ratings, the 1–9 rating scale was divided into 3 categories, where the ‘low’ category was defined as scores less than 4, ‘neutral’ category was defined as scores 4–6, and the ‘high’ category was defined as a score of greater than 6. For ratings of valence, low scores indicate unpleasantness and high scores indicate pleasantness. For ratings of arousal, low scores indicate calmness and high scores indicate excitedness. Mean responses to each sound were assigned to one of three categories for valence and one of three categories for arousal. The number of sounds whose category assignment was different for rms- and peak-level scaling approaches was calculated for ratings of valence and arousal separately.

Finally, to evaluate the relationship between a sounds’ acoustical properties and subjective ratings, correlations were conducted between ratings of valence (or arousal) and a sound’s rms level (within peak-scaled stimuli) or a sound’s peak level (within rms-scaled stimuli). This approach allows the exploration of the role of the alternative level cue when one level cue was normalized (rms- or peak- level) on subjective ratings of valence or arousal. Correlations were conducted using the *cor.test* function in base R; each of 4 potential relationships were explored using a separate correlations: 1) ratings of valence and rms level within peak-scaled sounds, 2) ratings of valence and peak level within rms-scaled sounds, 3) ratings of arousal and rms level within peak-scaled sounds, and 4) ratings of arousal and peak level within rms-scaled sounds.

## Results

### Ratings of valence

Analysis revealed a significant main effect of scaling approach (F[1, 4182] = 23.39, **p* *< .00001, η_p_^2^ = .01). Ratings of valence of the rms-scaled sound were approximately 0.20 points higher than the ratings of valence of the peak-scaled sounds (estimated marginal mean = 4.83 [rms-scaled] and 4.63 [peak-scaled]). Ratings of valence in the rms-scaled condition as a function of ratings of valence in the peak-scaled condition are displayed in Fig 2. Note that this trend is visible in the figure, where many of the points fall above the horizontal diagonal line, suggesting that the same stimuli are given different ratings based on the scaling approach utilized.

Also in Fig 2 is the visualization of the changes in category. Colored symbols indicate sounds that changed nominal categories between rms- and peak-scaling approaches. Of the 120 tokens, 103 were in the same valence category regardless of the amplitude-scaling approach used to present the sounds. However, 9 tokens which were categorized as ‘unpleasant’ with the peak-scaling approach were categorized as ‘neutral’ with the rms-scaling approach. Furthermore, 6 tokens categorized as ‘neutral’ in the peak-scaling approach were categorized as ‘pleasant’ with the rms-scaling approach, and 2 tokens categorized as ‘neutral’ with the peak-scaling approach were categorized as ‘pleasant’ with the rms-scaling approach. Combined, these results indicate that there was a main effect of amplitude-scaling approach on ratings of valence; ratings were higher with the rms- approach than the peak-scaling approach. However, the effect was small enough that only 14% tokens (17 out of 120) would be interpreted differently in the rms- and peak-scaling approaches. Note that none of the tokens changed 2 nominal categories (e.g., from pleasant to unpleasant or vice versa).

**Fig 3:**
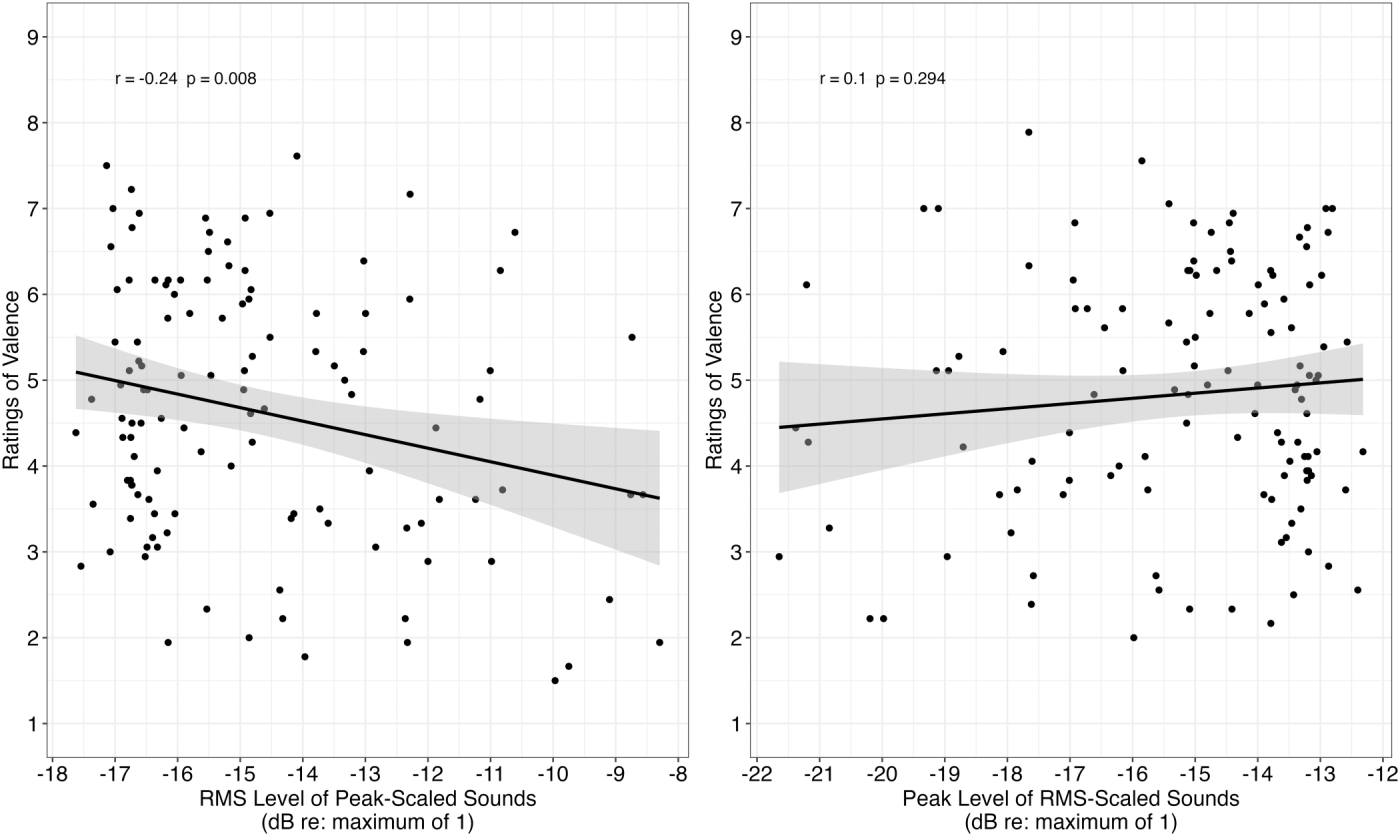
Mean ratings of valence as a function of rms level (among peak-scaled sounds; left panel) and as a function of peak level (among rms-scaled sounds; right panel). The relationship between ratings of valence and rms level is statistically significant, whereas the relationship between ratings of valence and peak level is not statistically significant.

Correlation analysis revealed rms level within peak-scaled sounds was significantly related to ratings of valence (Pearson correlation = −.24, t[118] = −2.70, **p* *= .008; left panel Fig 3). However, there was no significant relationship between a sound’s peak level and ratings of valence among tokens that were rms-scaled (Pearson correlation = .10, t[118] = 1.05, **p* *= .294; right panel Fig 3). These findings indicate that rms level is related to ratings of valence with a peak-scaling approach, but peak level is not related to ratings of valence with an rms-scaling approach.

### Ratings of arousal

**Fig 4:**
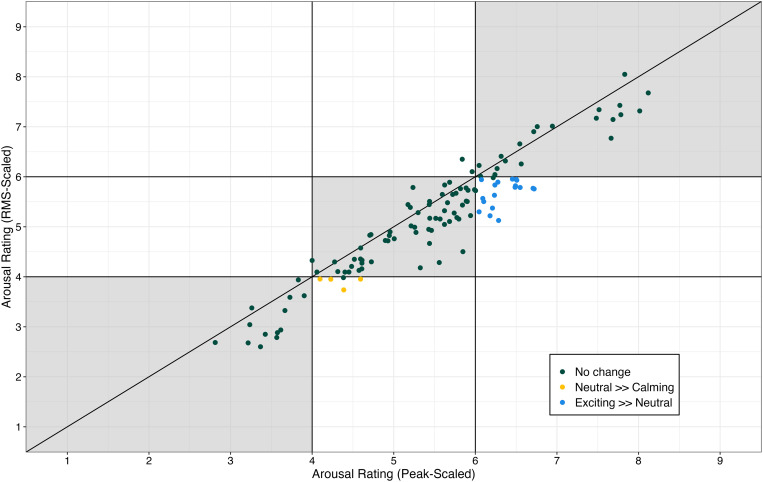
Mean rating of arousal of tokens when they were rms-scaled compared to when they were peak-scaled. Each dot represents an individual sound. Shaded regions indicate ratings that did not change nominal category between scaling approaches. Yellow dots indicate sounds that were nominally ‘neutral with the peak-scaling approach but were nominally ‘calming’ with the rms-scaling approach. Blues dots indicate sounds that were nominally ‘exciting’ with the peak-scaling approach but were ‘neutral’ with the rms-scaling approach*.*

Analysis revealed a significant main effect of scaling approach (F[1, 4421] = 23.41.56, **p* *< .00001, η_p_^2^ = .01). Ratings of arousal of the rms-scaled sounds were approximately 0.26 points lower than the ratings of arousal of the peak-scaled sounds (estimated marginal mean = 5.18 [rms-scaled] and 5.44 [peak-scaled]). Ratings of arousal in the rms-scaled condition as a function of ratings of valence in the peak-scaled condition are displayed in Fig 4. Note that this trend is visible in the figure, where many of the points fall below the horizontal diagonal line. Also evident in Fig 4 is that the lower ratings of arousal changed the nominal arousal category for 22 tokens; 18 changed from being ‘exciting’ to ‘neutral’ and 4 changed from ‘neutral’ to ‘calming.’ These data indicate that 18% of the tokens would be interpreted differently (less arousing) with the peak-scaling approach than the rms-scaling approach.

**Fig 5:**
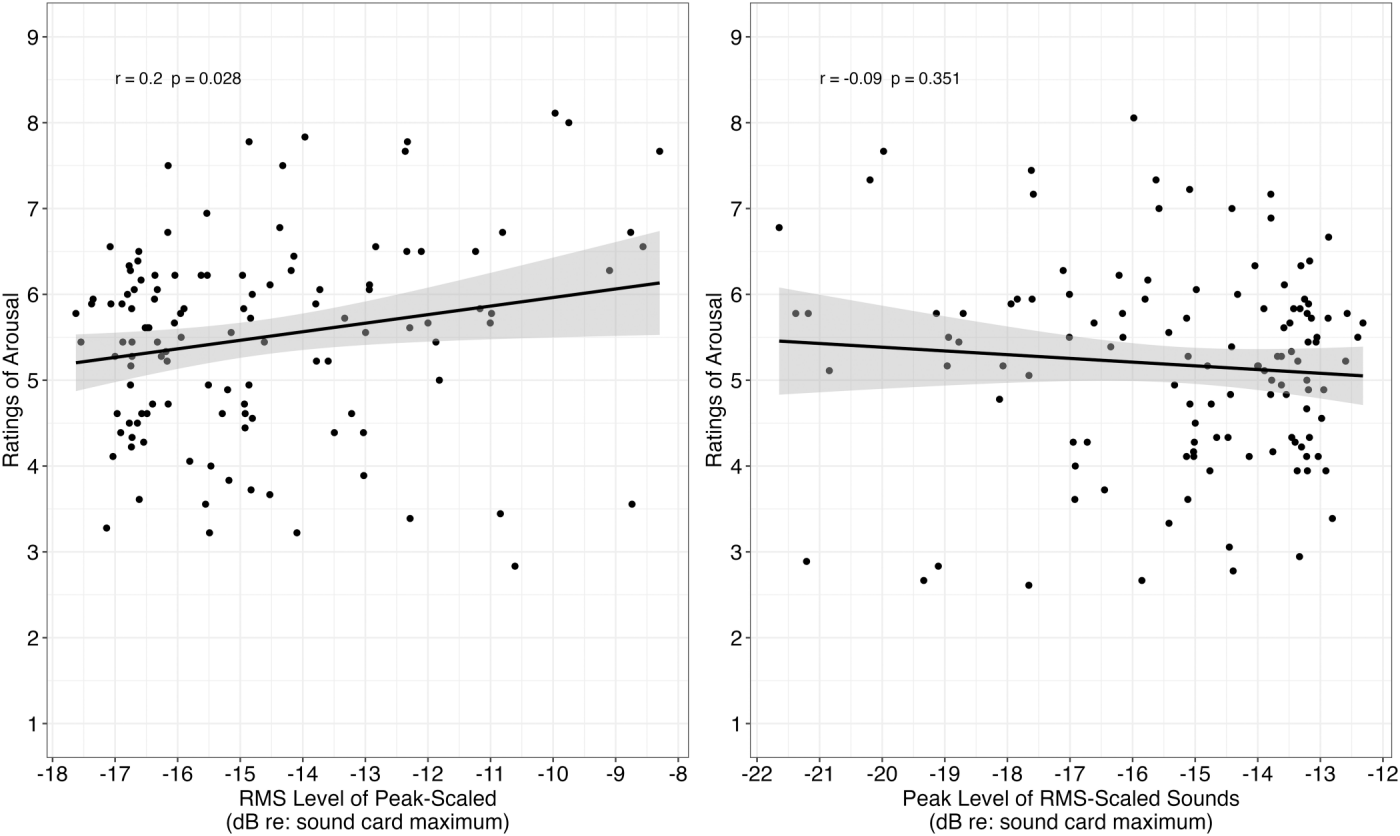
Mean ratings of arousal as a function of rms level (among peak-scaled sounds; left panel) and as a function of peak level (among rms-scaled sounds; right panel). The relationship between ratings of arousal and rms level was statistically significant, whereas the relationship between ratings of arousal and peak level was not statistically significant.

Correlation analysis revealed rms level within peak-scaled sounds was significantly related to ratings of arousal (Pearson correlation = 0.20, t[118] = 2.22, **p* *= .028; left panel Fig 5). However, there was no significant relationship between a sound’s peak level and ratings of arousal among sounds that were rms-scaled (Pearson correlation = −.08, t[118] = −0.86, **p* *= .351; right panel Fig 5). These findings indicate that rms level was related to ratings of arousal within a peak-scaling approach, but peak level was not related to ratings of arousal within an rms-scaling approach.

## Discussion

The purpose of this study was to evaluate the effects of amplitude-scaling approach (rms, peak) on emotional responses, specifically ratings of valence and arousal. The data analyzed in this study was from adults with self-reported normal hearing who passed attention checks during the online testing; they also provided ratings of valence and arousal of validated non-speech sounds that were consistent with normative data of those sounds. Within this group of participants, the choice of amplitude scaling approach affected both ratings of valence and ratings of arousal. Specifically, with an rms-scaling approach, ratings of valence were approximately 0.20 higher than ratings of valence with the peak-scaling approach. In addition, ratings of arousal were approximately 0.26 points lower with an rms-scaling approach compared to a peak-scaling approach. These findings indicate that, in general, tokens were rated as more pleasant and less exciting when they were rms-scaled than when they were peak-scaled. However, the change in amplitude-scaling approach nominally affected fewer than 20% of tokens. Out of 120 sounds, 103 and 98 sounds had ratings of valence and arousal, respectively, that did not change nominal category between amplitude-scaling approaches.

### Role of acoustics

In order to better understand the role of acoustics on ratings of valence and arousal, correlations were conducted between ratings of valence (or arousal) and level for the alternate level cue within a scaling approach. With the peak-scaled sounds, the amplitude naturally varies and the rms-level could be higher for some sounds than other sounds. As displayed in Fig 1, sounds with relatively steady amplitude would have higher overall rms values for peak-scaled sounds than for rms-scaled sounds. Correlation analysis revealed that the amplitude of a sound affected ratings of valence and arousal, but only within the peak-scaled sounds. It is not surprising that ratings are related to rms level. Previous work has demonstrated that increasing the overall level results in lower ratings of valence for peak-scaled sounds [20,21]. The current work extends previous findings to show that the relationship between rms level and ratings of valence is statistically significant within individual sounds. Also consistent with previous literature [59–63], and with the notion that arousal reflects degree of activation of an emotion [17–19], sounds with higher rms were rated as more exciting than those with lower rms.

These rms-level related differences could account for the general differences in ratings between the two scaling approaches, depending on the level-setting strategy of participants. The overall rms was approximately 12 dB higher for the peak-scaled sounds (−14.7 dB relative to a maximum of 1) than the rms-scaled sounds in this study (−26.9 dB relative to a maximum of 1). If participants used the same presentation level setting for both sets of tokens, it would explain the lower ratings of valence and higher ratings of arousal for the peak-scaled than the rms-scaled sounds. Participants were encouraged to individually set the level for each block prior to testing, but the absolute level of the system, and their volume settings, were not accessible for analysis.

### Research implications

The results of this study suggest the valence ratings in response to sounds used for emotion perception and distraction tasks were affected by amplitude-scaling approach. This indicates that the methodological choice of scaling sounds by peak or rms level might affect interpretation of emotional responses to non-speech sounds. This is especially important considering use of the peak- or rms-scaling strategies are mixed in the literature, with some using rms-scaling strategies [e.g., 55,56,57] and some using peak-scaling strategies [20,21]. Understanding that the amplitude-scaling approach has the potential to affect ratings of valence and arousal has the potential to make data synthesis across studies difficult, if different amplitude-scaling approaches are used.

However, it is important to note the scale of the differences in the current study is small. The 0.2-point rating difference represents a 2-percentage point difference on the 9-point scale used for facilitating ratings of responses. Furthermore, only 14% and 18% of sounds changed nominal categories. In addition, none of the sounds changed more than 1 nominal category. Therefore, although the effect of amplitude-scaling approach was statistically significant and affected some sounds, the effects are generally small.

Importantly, the choice of amplitude-scaling approach not only affects overall ratings of valence and arousal (~0.2 points lower and higher, respectively, with peak-scaled sounds), but also the extent to which amplitude was related to emotion perception. With the peak-scaled sounds, the sounds were matched on one dimension (peak level), but the rms varied between sounds and served as a cue for ratings of valence and emotion. Conversely, the rms-scaled sounds were matched on one dimension (rms level), but the variability in peak level across sounds did not serve as a cue for ratings of valence and emotion. Therefore, in order to preserve some of the individual level variability that contributes to emotion perception of non-speech sounds, it might be beneficial to use a peak-scaling approach. Although ratings of valence and arousal might be generally lower and higher, respectively, than if an rms-scaling approach was used, the peak-scaling allows level to be a cue for ratings of valence and arousal. Conversely, if eliminating level differences is desirable and a researcher wants to explore other cues that might contribute to emotion perception of non-speech sounds, then an rms-scaling approach might be preferable.

### Study limitations

There are several study limitations worth mentioning. First, the testing occurred remotely. The unsupervised testing precludes full confidence that participants were not distracted, although attention checks and forced rest breaks serve to improve the chance that study participants were authentic in their responses. Because it was an online study, it is also possible some of the participants had audiometric hearing loss, despite self-reported normal hearing. This could potentially be problematic because people with hearing loss have been shown to provide reduced range of ratings in emotion perception tasks [20]. Fortunately, previous work demonstrates that online testing can be a viable alternative to laboratory testing for hearing-related tasks generally [79] and for measuring emotional responses to non-speech sounds specifically [23,70]. Future work is warranted to evaluate if the results of the current study replicate in a laboratory setting.

Future work is also warranted to determine how these findings will translate to different stimuli. These results are specific to the 120 tokens from a corpus of non-speech sounds, all of them 0.5 seconds in duration. The emotion perception literature often uses an rms-scaling approach with speech stimuli [e.g., 40, 46], although speech corpora are not exclusively rms-scaled [e.g., 80]. It is possible the effects of rms- versus peak-scaling would be smaller with speech tokens because speech tends to have a narrower amplitude dynamic range than other sounds, such as music [e.g., 81]. However, non-speech sounds are inherently variable [53] and therefore current results will only apply to sounds with similar temporal dynamics as the stimuli in the current study. Future work is warranted to evaluate amplitude scaling approach differences in the study of emotion perception with speech stimuli.

Finally, the study focused on only two amplitude scaling approaches. There are other approaches that are standardized that could have been used in this study, such as the European Broadcasting Union Recommendation R128, which has been suggested to be superior to either peak- or rms-level scaling approaches [68]. Although such scaling approaches have not been reported in the study of emotional responses to non-speech sounds, it is possible the use of a widely accepted standard would be beneficial in the field. Importantly, neither peak- nor rms-level scaling account for auditory sensitivity of listeners. The conclusions about level as a cue for ratings of valence and arousal are not based on predicted loudness models [82–84]. Both scaling approaches ignore the spectrotemporal characteristics of human hearing and thus equate only level, not loudness. Future work is warranted to evaluate the extent to which loudness cues for emotional responses contribute to the conclusions of this study.

## Conclusions

The purpose of this study was to evaluate the effects of amplitude-scaling approach (rms, peak) on emotional responses, specifically ratings of valence and arousal. The results reveal ratings of valence were lower, and ratings of arousal were higher, in response to peak-scaled sounds than in response to rms-scaled sounds, although the effects were generally small and nominally affected fewer than 20% of the tokens. Within the peak-scaled sounds, those with higher rms values also had ratings of valence that were lower (less pleasant) and ratings of arousal that were higher (more exciting) than for sounds with lower rms values. This finding suggests that peak-scaling approaches preserve some of the individual variability in level that can affect ratings of valence and arousal. Investigators studying emotional responses to non-speech sounds should consider a scaling approach that fits their research agenda, specifically whether variability in level across scaled-tokens is important (preserved with peak-scaling) or whether reducing overall level as a cue is important (with rms-scaling). In addition, care should be used when synthesizing valence and arousal findings across studies with different amplitude-scaling approaches because the chosen approach affects not only overall ratings, but also the acoustic cues listeners are using to make ratings of valence and arousal.
